# Bibliographical Notices

**Published:** 1844-04-06

**Authors:** 


### BIBLIOGRAPHICAL NOTICES.

*A Dictionary of Medical Science, containing a concise account of the various subjects and terms, with the French and other Synonymes; Notices of Climate, and of Celebrated Mineral Waters; Formulæ for various Officinal and Empirical Preparations,* &c. By ROBLEY DUNGLISON, M.D., Professor of the Institutes of Medicine, &c., in Jefferson Medical College of Philadelphia, &c. &c. *Fourth Edition, extensively modified and enlarged.* Philadelphia: Lea & Blanchard. 1844 Royal Octavo, pp. 771.

Although less than two years have elapsed since the publication of the previous Edition, the Author has found it requisite to add to the present not fewer than *two thousand* subjects and terms; many of which have been introduced into medical terminology in consequence of the progress of the science. This fact shows how necessary it is that such a book should undergo frequent revisions. The only other work of the kind (Hooper's) in the market, has been stereotyped, according to our recollection, some twenty or more years, and consequently contains none of the terms introduced by Laënnec and others, expressive of the physical signs of diseases of the chest, names of modern pharmaceutical agents and combinations, besides thousands of other terms now in frequent use. We hope that neither the Author nor his Publishers, will ever be induced to stereotype this work. So long as the science is progressive, so long we shall have new terms to represent the improvements and alterations that occur, and a dictionary to be useful must embrace them. The present Edition of Dunglison's Medical Dictionary is not only more complete than any that has preceded it, but it is the very best work of the kind extant. We cannot conceive how any member of the profession can do without it.

---

*A Treatise on the Diagnosis and Treatment of Diseases of the Chest, Diseases of the Lung, and Windpipe.* By WILLIAM STOKES, M.D., M.R.I.A., Physician to the Meath Hospital and County of Dublin Infirmary, &c. &c. Second Edition. *With an Introduction and numerous Notes.* By the American Editor. Philadelphia, Edward Barrington and George D. Haswell. 1844. Octavo, pp. 528.

This, we believe, is the Second *American* Edition of the work of Dr. Stokes on the Chest, it having appeared, or at least the greater part of it, several years ago in the "Library" conducted by Dr. Dunglison. The present Edition, however, is enriched by the labours of the Editor, (Dr. Bell,) whose additions consitute, in fact, no inconsiderable portion of the book.

Dr. Stokes is so well known as a judicious and eminently practical writer, that it is scarcely necessary to speak of the present treatise further than to indicate the particular subjects which it embraces. A leading object in the preparation of this work, seems to have been to point out the close connexion of the study of physical signs with that of symptoms, in affections of the respiratory organs, so as to illustrate their mutual bearing on diagnosis, and remove the unjust opprobrium thrown on the advocates of auscultation, that they neglect the study of symptoms. The author has endeavoured to simplify the subject as far as possible, and "to adapt the work to the wants of the practical man, always assuming that he is familiar with the groundworks of the subject—with the characters and causes of physical signs, as originally taught by Laennec, and more recently investigated in the works of Forbes, Williams and Clarke." In other words, he has not gone at any length into the characters of physical signs, but rather into the art of reasoning justly upon them. Herein, we think, the text is deficient. We hold with Dr. Johnson, that to be complete, a book should contain all that the reader may wish to know on the subject on which it is written. This deficiency, however, is supplied in a great measure by the remarks of the Editor, and his judicious selections from Walshe and others, contained in his introductory chapter.

The work is divided into a number of SECTIONS, treating severally of the diagnosis of thoracic diseases; diseases of the mucous membrane; diseases of the larynx and trachea; pneumonia; gangrene of the lung; perforating abscess of the lung; cancer of the lung; tubercle of the lung; and diseases of the pleura; beside the matter supplied by the Editor.

Altogether, this is one of the most useful of the publications that have recently issued from the press,—one that should be studied by every man who presumes to treat the class of diseases on which it is written. It is delightful, indeed, to contemplate the sound pathology, and rational therapeutics which it inculcates;—so different from the swelling egotism, the groundless assertions, the illogical conclusions, and often empirical treatment, recommended in some of the modern works on these and other important medical subjects.

